# SOX6 Downregulation Induces *γ*-Globin in Human *β*-Thalassemia Major Erythroid Cells

**DOI:** 10.1155/2017/9496058

**Published:** 2017-11-28

**Authors:** Jing Li, Yongrong Lai, Jun Luo, Lin Luo, Rongrong Liu, Zhenfang Liu, Weihua Zhao

**Affiliations:** Department of Hematology, The First Affiliated Hospital of Guangxi Medical University, Nanning, Guangxi 530021, China

## Abstract

**Background:**

Fetal hemoglobin (HbF; *α*_2_*γ*_2_) is a potent genetic modifier of the severity of *β*-thalassemia and sickle cell anemia. Differences in the levels of HbF that persist into adulthood affect the severity of sickle cell disease and the *β*-thalassemia syndromes. Sry type HMG box (SOX6) is a potent silencer of HbF. Here, we reactivated *γ*-globin expression by downregulating SOX6 to alleviate anemia in the *β*-thalassemia patients.

**Methods:**

SOX6 was downregulated by lentiviral RNAi (RNA interference) in K562 cell line and an* in vitro* culture model of human erythropoiesis in which erythroblasts are derived from the normal donor mononuclear cells (MNC) or *β*-thalassemia major MNC. The expression of *γ*-globin was analyzed by qPCR (quantitative real-time PCR) and WB (western blot).

**Results:**

Our data showed that downregulation of SOX6 induces *γ*-globin production in K562 cell line and human erythrocytes from normal donors and *β*-thalassemia major donors, without altering erythroid maturation.

**Conclusions:**

This is the first report on *γ*-globin induction by downregulation of SOX6 in human erythroblasts derived from *β*-thalassemia major.

## 1. Introduction

Beta-thalassemia syndromes are a group of hereditary blood disorders characterized by reduced or absent *β*-globin chain synthesis, resulting in anemia [1]. Clinical evidence indicates that elevated fetal hemoglobin (HbF; *α*_2_*γ*_2_) production mitigates the severity of *β*-thalassemia [2]. Patients with two *β*-thalassemia alleles combined with hereditary persistence of fetal hemoglobin (HPFH) allele have milder clinical presentations [3, 4]. Therefore, HbF is a major modifier of the severity in *β*-thalassemia.

The predominant globin in normal infants is *γ*-globin, which switches to *β*-globin few months after birth, by a process known as the fetal-to-adult hemoglobin switch [5]. Thus, understanding the molecular mechanisms responsible for *γ*- to *β*-globin switching and increasing *γ*-globin gene expression as a potential therapy for *β*-hemoglobinopathy is critical. Recent studies on *γ*-globin expression have shed new light on this complex regulatory process.

Sry type HMG box (SOX6) is a chromatin-associated protein (member of the SRY-related high mobility group (HMG) box transcription factors) that binds and induces a marked bending of DNA [6]. Recently, SOX6 was shown to stimulate erythroid cell survival, proliferation, and terminal maturation during definitive murine erythropoiesis [7, 8]. In 2006, SOX6 was first identified as a novel and crucial silencing factor of *ε*y-globin in mice. SOX6 acts as a repressor by directly binding to the *ε*y promoter [9]. SOX6 and BCL11A, the latter being a master repressor of *γ*-globin [10–12], cooccupy the human *β*-globin cluster along with GATA1 and cooperate in silencing *γ*-globin transcription in adult human erythroid progenitors [13]. SOX6 could be a potentially promising target for *γ*-globin induction. There is no report yet on downregulation of SOX6 to induce *γ*-globin expression in human erythroblasts derived from *β*-thalassemia major.

Taken together, SOX6 was chosen as target mediator to induce *γ*-globin expression in human erythroblasts derived from *β*-thalassemia major.

## 2. Materials and Methods

### 2.1. Cell Culture

Mononuclear cells (MNC) were isolated from normal human peripheral blood (PB) and *β*-thalassemia major patients bone marrow (BM) by density gradient centrifugation with Ficoll-Hypaque solution (Solarbio life sciences). The cells were incubated in a one-stage liquid culture system (Table 1).

Three normal donors and three *β*-thalassemia major patients were analyzed. Each donor's cells were separately cultured. Cells were incubated at 37°C in a humidified atmosphere of 5% CO_2_ and maintained at a density of 5 × 10^5^ cells/ml by supplementing cultures with fresh media every four days. Cell numbers and viability were determined by trypan blue exclusion. Cell morphology was assessed by phase-contrast microscopy (Olympus CKX41) and Wright-Giemsa staining of cytocentrifuge preparations.

The information on the cultured cells is shown in Supplementary Figures 1–5.

We also tried to purify CD34^+^ cells from MNCs by immunomagnetic beads separation technique. The harvested CD34^+^ cells were cultured till the end stage but did not reach enough numbers to extract mRNA or protein. So, MNC was chosen for culture.

K562 cells (gift from Institute of Hematology, Chinese Academy of Medical Sciences) were incubated in DMEM (Invitrogen) with 10% fetal calf serum (Invitrogen).

There were three groups of cells: ① control group (C), untreated; ② control vector group (CV), treated with control lentiviruses; and ③ sh-RNA group (SH), treated with lentiviruses containing target shRNA.

The research protocol was approved by the ethics committee of Guangxi Medical University in China.

### 2.2. Lentiviral shRNA and Lentivirus Production and Transduction

Lentiviral shRNA constructs in the pLKO.1-PURO vector were purchased from Sigma® Life Science. The pLenti4/BLOCK-IT™-DEST vector (gift from Tsinghua University in China) was used to produce control lentiviruses. We selected two different shRNA sequences for SOX6 and then chose the sequence with the most obvious inhibitory effect. The shRNA sequence is listed in Table 2.

293FT cells were transduced with 10 *μ*g pLKO.1-PURO/pLenti4/BLOCK-IT-DEST vector, 15 *μ*g VSVG 1.0 vector, and 15 *μ*g Δ8.9 vector. The cells were incubated with 20 ml Opti-MEM (Gibco) and 120 *μ*l Lipofectamine 2000 (Invitrogen) solutions for six hours, after which the media was replaced. After 72 hours, media containing virus was filtered and collected. The virus titer was determined by crystal violet staining method and was found to be 5 × 10^6^ TU/ml.

For transduction, MNCs were grown for 0 and 24 hours at a concentration of 5 × 10^5^ cells/ml in media incubated overnight with vector particles (multiplicity of infection [MOI], 10). Cells at end stage of differentiation (the 15th day) were harvested to extract mRNA.

K562 cells were incubated with the viruses at a concentration of 5 × 10^5^ cells/ml (MOI, 10) overnight. Transduced cells were selected by puromycin. On the 12th day of culture, the cells were harvested to extract mRNA and protein.

### 2.3. Western Blot

Whole cell extracts were obtained by lysis in RIPA buffer (Table 3). A total of 60 *μ*g of protein was diluted with 6x SDS-loading buffer, denatured by boiling for five minutes, and resolved on a 10% or 12% SDS-PAGE gel. Proteins were then transferred to a PVDF membrane by wet transfer procedure. The membrane was incubated with relevant primary antibodies overnight at 4°C. This was followed by incubation with anti-rabbit or anti-goat horseradish peroxidase- (HRP-) conjugated secondary antibodies (1 : 3000 dilutions) in 5% milk in 1x TBS and 0.1% Tween-20 for one hour.

Antibodies against *γ*-hemoglobin (ab137096) was purchased from Abcam. Antibody against SOX6 (A303-553A) was purchased from Bethyl. Antibody against GAPDH (60004-I-Ig) was purchased from Proteintech. Molecular weights and dilution ratios of primary antibodies are listed in Table 4.

### 2.4. Flow Cytometry

Cells (50–100,000) at the end stage of differentiation (the 15th day) were rendered into single-cell suspensions for flow cytometric analysis. Cells were tested for expression of cell surface receptors with antibodies specific for CD235a (BD Biosciences) conjugated to fluorescein isothiocyanate on a FACS Calibur System (BD Biosciences) using CellQuest analysis software (BD Biosciences). Isotype control was also used. CD235a is a type I transmembrane sialoglycoprotein that is expressed on human erythrocytes and is useful for their identification and characterization.

### 2.5. RNA Isolation and qPCR

RNA was isolated using the RNeasy mini kit (Qiagen) according to the manufacturer's protocol. cDNA was synthesized with the iScript cDNA synthesis kit (Bio-Rad). Quantitative real-time PCR (qPCR) was performed using the Faststart Universal SYBR Green (Roche). Amplification reactions were performed with an Optical IQ Thermal Cycler (Bio-Rad) with the following conditions: 95°C for 10 minutes, followed by 40 cycles of 95°C for 15 seconds and 60°C for 60 seconds. All reactions were performed in triplicate. Relative gene expression was quantified using the ΔΔCt method. Target gene expression was normalized to GAPDH expression, unless indicated otherwise. The relative values of *γ*-globin and *β*-globin mRNA for each sample were calculated using the Ct method using GAPDH transcript signal as an internal control. Primers used in this study are listed in Table 5.

### 2.6. Statistical Analysis

Statistical analysis of gene expression data obtained from qPCR was performed with the one-way ANOVA test and SPSS16.0 statistical software. All results are means ± SD from three independent experiments. A value of two-sided *p* less than 0.05 was considered statistically significant.

## 3. Results

K562 cell line is an erythroleukemic human “fetal” erythroid cell line, expressing *γ*-globin gene dominantly and a little *β*-globin gene. First, we selected this cell line to observe the effect of downregulation of SOX6 on *γ*-globin expression.

We then repeated the procedure in primary adult erythroid cells from normal donors or patients with *β*-thalassemia major. Human cytokine-mobilized adult PB MNCs from 3 normal donors and human *β*-thalassemia major bone marrow MNCs from 3 patients were transduced with vector. Three of the thalassemia major patients were compound heterozygous for a *β*^0/^*β*^+^ mutation. Each donor's cells were separately cultured. MNCs were placed in liquid cultures that promote unilineage erythroid differentiation. Erythroid cell differentiation was analyzed by cell morphology (Wright-Giemsa staining), CD235a expression (qPCR and flow cytometry), and hemoglobin gene expression (qPCR). At the end of the culture period, about 90% of the cultured cells were erythrocytes (the information on the cultured cells is shown in Supplementary Figures 1–5). Taken together, these data show effective erythropoiesis in vitro.

Downregulation of SOX6 expression in K562 cells and primary adult erythroid cells from normal donors or patients with *β*-thalassemia major led to elevated *γ*-globin expression.

**Figure 1:**
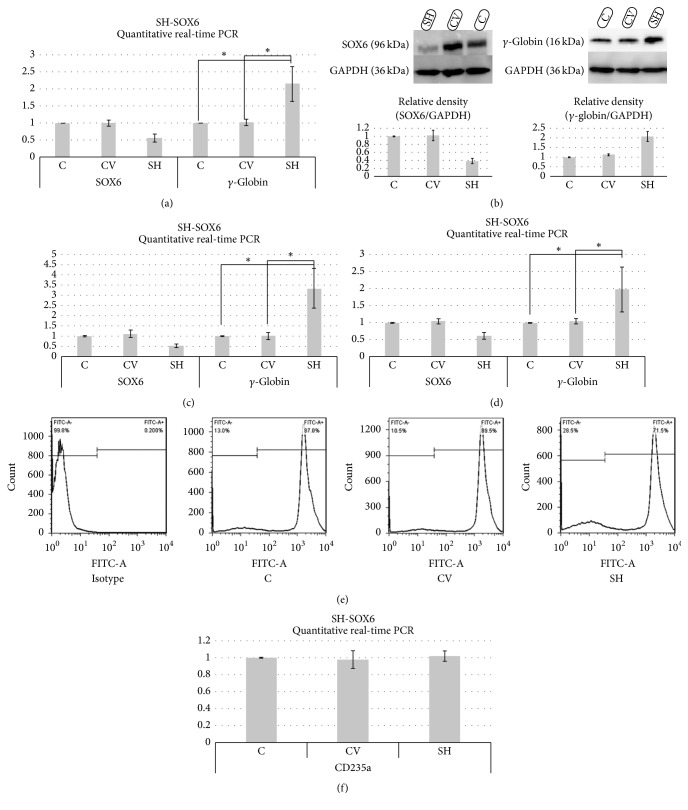
(a) qPCR analysis showed that downregulation of SOX6 expression in K562 cells induces *γ*-globin production. (b) Western blot analysis showed that downregulation of SOX6 expression in K562 cells induces *γ*-globin production. (c) qPCR analysis showed that downregulation of SOX6 expression in primary adult erythroid cells from normal donors induces *γ*-globin production. (d) qPCR analysis showed that downregulation of SOX6 expression in primary adult erythroid cells from patients with *β*-thalassemia major induces *γ*-globin production. (e) Flow cytometry analysis showed that in cells from patients with *β*-thalassemia major transduced with SOX6 shRNA, there are no obvious changes in CD235a expression as compared to the control group or control vector group. (f) qPCR analysis showed that, in primary adult erythroid cells transduced with SOX6 shRNA, there are no significant differences in CD235a expression as compared to the control group or control vector group. C group: control; CV group: control vector; SH group: shRNA. ^*∗*^*p* < 0.01.

In K562 cells, the effects of SOX6 knockdown on altering the expression of *γ*-globin were analyzed by qPCR and WB. As shown in Figure 1(a), *γ*-globin mRNA expression showed 2.15-fold increase in shRNA group compared to control group. WB analysis also demonstrated elevated *γ*-globin protein level in shRNA group, as shown in Figure 1(b).

In primary adult erythroid cells, the effects of SOX6 knockdown on altering the expression of *γ*-globin were analyzed only by qPCR on day 15 of erythroid differentiation due to the reasons mentioned above. As shown in Figure 1(c), *γ*-globin mRNA expression showed 3.35-fold increase in shRNA group compared to control group in primary adult erythroid cells from normal donors. And in erythroid cells from patients with *β*-thalassemia major, *γ*-globin mRNA expression showed 1.97 fold increase in shRNA group compared to control group, as shown in Figure 1(d).

In shRNA group, there were significant differences in *γ*-globin expression as compared to the control group or control vector group (*p* < 0.01) both in K562 cells and in primary adult erythroid cells from normal donors or patients with *β*-thalassemia major (Figures 1(a), 1(c), and 1(d)).

Erythroid differentiation was not affected by SOX6 knockdown as all cultures showed similar levels of CD235 positivity, as shown in Figures 1(e) and 1(f). In primary adult erythroid cells transduced with SOX6 shRNA, there were no significant differences in CD235a expression as compared to the control group or control vector group (Figure 1(f)).

Each normal donor and *β*-thalassemia major patient data were listed in Table 6.

## 4. Discussion

*β*-thalassemia remains a major global health challenge, particularly in the developing countries. Its clinical management includes supportive care and iron chelation. Bone marrow transplantation provides a genetic cure but cannot be widely applied [14]. Several important natural observations demonstrated that the severity of *β*-thalassemia could be ameliorated via increased production of *γ*-globin [2–4]. Modifier genes of *γ*-globin and hemoglobin switching have been long studied due to their potential usage in developing targeted therapeutic approaches for *β*-thalassemia [5].

SOX6 is a member of the SOX (Sry-type HMG box) family of transcription factors, characterized by the presence of an HMG domain that recognizes the minor groove on DNA. The binding of SOX proteins to DNA forces it to bend at about 75°, introducing local conformational changes. The ability of SOX proteins to bind in close proximity to other transcription factors and to distort DNA suggests that they can act as “architectural proteins,” possibly by promoting the assembly of biologically active multiprotein complexes. These complexes, in turn, mediate the interactions between distant chromatin domains, bringing together promoter/enhancer regions, finally assembling the “chromatin hubs” that control gene expression regulation [15–17]. The SOX6 transcription factor plays critical roles in various cell types, including erythroid cells [18]. A potential role for SOX6 in globin gene regulation was first recognized by analysis of the SOX6-deficient mouse. At the fetal liver stage, expression of mouse embryonic *β*-like globins (*ε*y and *β*h1) was dramatically elevated in the SOX6-deficient p100H mouse [9]. BCL11A is a critical mediator of *γ*- to *β* globin switching in mammals. Induction of *γ*-globin by downregulation of BCL11A was demonstrated in primary human adult erythroid progenitors and mice [10–12]. A recent study suggests that transcriptional silencing of *γ*-globin by BCL11A involves long-range interactions and cooperation with SOX6. BCL11A mediates silencing of *γ*-globin genes through both long-range interaction within the human *β*-globin cluster and local interactions with the chromatin-associated SOX6 proteins at the proximal promoters of the *γ*-globin genes [13]. SOX6, by interacting with *γ*-proximal promoters, may help recruit BCL11A to the proximal regions of the *γ*-genes during hemoglobin switching [13]. SCF-mediated *γ*-globin gene expression in adult human erythroid cells is also associated with SOX6 downregulation [19]. Taken together, SOX6 may also be a potentially promising target for HbF induction.

Our data showed that downregulation of SOX6 induced production of *γ*-globin in K562 cells and primary adult erythroid cells from normal donors or patients with *β*-thalassemia major. Downregulation of SOX6 did not impair differentiation of erythroblast, which is consistent with previous report [13]. This is the first report on the induction of *γ*-globin by downregulation of SOX6 in primary adult erythroid cells from patients with *β*-thalassemia major.

Besides its role in hemoglobin regulation, SOX6 also plays an important role in the development of the central nervous system [20], cartilage [21], and muscle [22]. Murine SOX6 null mutants (p100H) show delayed growth, myopathy, and atrioventricular heart block and die within 2 weeks following birth [23]. Previous studies on the role of SOX6 in hemoglobin regulation have not revealed any impact of its downregulation on other systems.

## 5. Conclusions

So, SOX6 is a critical regulator of *γ*-globin. Our data and previous studies suggest that this transcription factor is promising targets to influence *γ*-globin expression for alleviating anemia in *β*-thalassemia. Given that SOX6 is not erythroid specific, its safety and exact roles in *γ*-globin regulation need to be intensively explored.

## Figures and Tables

**Table 1:** One-stage liquid culture system.

Reagent	Company	Concentration
RPMI 1640	Hyclone	
Fetal calf serum	*Invitrogen*	30%
Erythropoietin	Kyowa Hakko Kirin Co., Ltd.	3^ ^units/ml
Dexamethasone	Sigma	10^−6 ^M
*β*-Estradiol	Solarbio life sciences	10^−4 ^M

**Table 2:** shRNA sequence.

shRNA targeting gene	shRNA sequence
SOX6	CCGGCGTGAGATAATGACCAGTGTTCTCGAGAACACTGGTCATTATCTCACGTTTTT

**Table 3:** RIPA buffer.

50 mM Tris	0.6057 g	1.51 g
150 mM NaCl	0.8766 g	2.19 g
0.5% NaDeoxycholate	0.5 g	1.25 g
1% NP40	1 ml	2.5 ml
0.1% SDS	0.1 g	0.25 g
pH to 8	dH2O to 100 ml	dH2O to 250 ml

**Table 4:** Dilution ratio of primary antibody.

Antibody name	Molecular weight	Dilution ratio
SOX6	96 kDa	1 : 1000
*γ*-Hemoglobin	16 kDa	1 : 1000
GAPDH	36 kDa	1 : 3000

**Table 5:** Primer sequence.

Name of locus	Primes (5′-3′)	Product size
SOX6	CTCCTGCAGCAACAGATCCA AGAGGAATCCCTGTTGGGCA	115 bp
*γ*-Hemoglobin	TCGCTTCTGGAACGTCTGAG GTAGACAACCAGGAGCCTTCC	157 bp
*β*-Hemoglobin	GTCTACCCTTGGACCCAGAGGTTC TGAGCCAGGCCATCACTAAAG	131 bp
GAPDH	GTCAGCCGCATCTTCTTT CGCCCAATACGACCAAAT	99 bp
CD 235a	TCCAGAAGAGGAAACCGGAGA AAAGGCACGTCTGTGTCAGG	195 bp

**Table 6:** Information of each donor or patient (SOX6).

	*γ*-Globin (fold increase)	SOX6 (percentage knockdown)	HbF (baseline)	Genotype
Donor 1	3.85	48.7%	1.1%	/
Donor 2	4.07	55.2%	0.8%	/
Donor 3	2.08	35.1%	2.3%	/
Patient 1	2.63	29.9%	31.3%	*β* ^0/^ *β* ^+^
Patient 2	2.08	36.0%	45.7%	*β* ^0/^ *β* ^+^
Patient 3	1.16	41.7%	65.6%	*β* ^0/^ *β* ^+^

Donor: health donor; Patient: *β*-thalassemia major patient; *γ*-globin (fold increase): fold increase in *γ*-globin gene expression in shRNA group compared to control group; SOX6 (percentage knockdown): percentage knockdown of SOX6; HbF (baseline): inherent baseline HbF percentage of each donor and patient; genotype: genotype of each *β*-thalassemia major patient.

## Supplementary Materials

Supplementary 1 Supplementary Figure  1: Representative cytospin images of proerythroblasts (Pro-E), basophilic erythroblasts (Baso-E), polychromatic erythroblasts (Poly-E), orthochromatic erythroblasts (Ortho-E), and mature erythrocytes.

Supplementary 2 Supplementary Figure  2: Proportion of erythrocytes in the differentiated cultured cells on day 5, day 10, and day 15, verified by cell count based on Wright-Giemsa-stained morphology (counts of cells include Pro-E, Baso-E, Poly-E, Ortho-E, and mature erythrocytes).

Supplementary 3 Supplementary Figure  3: Flow cytometry analysis showing CD235a expression in cultured cells after 15 days.

Supplementary 4 Supplementary Figure  4: qPCR showing CD235a expression in differentiated cultured cells on day 5, day 10, and day 15.

Supplementary 5 Supplementary Figure  5: qPCR showing *γ*/*β*-globin mRNA ratios in differentiated cultured cells on day 5, day 10, and day 15.
