# Supplementary material for: SOX6 Downregulation Induces γ-Globin in Human β-Thalassemia Major Erythroid Cells
Source: Biomed Res Int. 2017 Nov 28;2017:9496058. doi: 10.1155/2017/9496058 (PMC5733236; doi:10.1155/2017/9496058)

**Supplementary Figure 1.** Representative cytospin images of proerythroblasts (Pro-E), basophilic erythroblasts (Baso-E), polychromatic erythroblasts (Poly-E), orthrochromatic erythroblasts (Ortho-E) and mature erythrocytes.


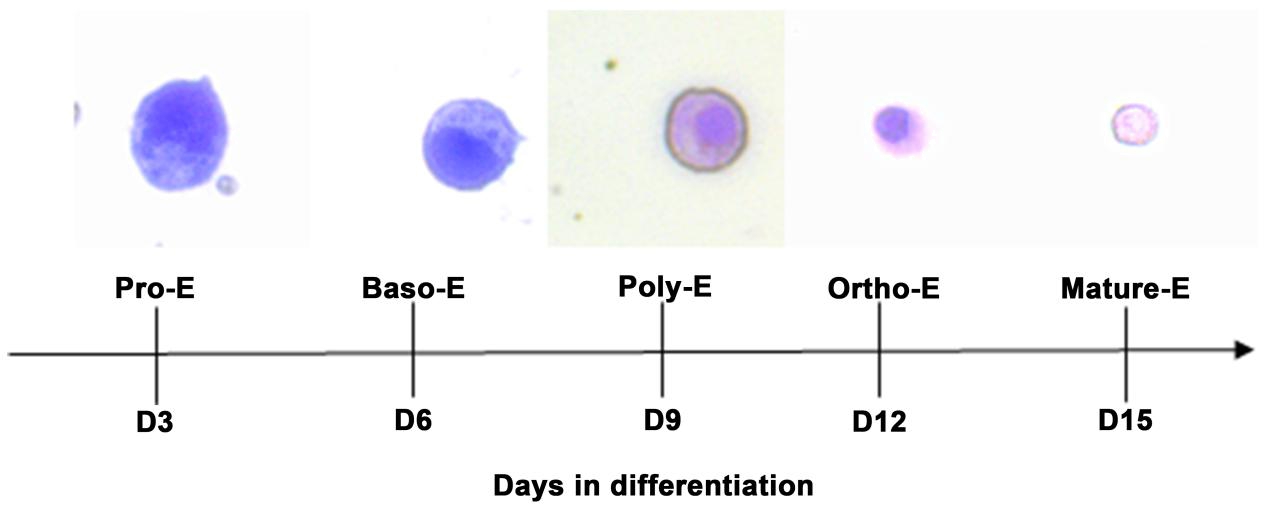

Supplement: Supplementary 1 — Supplementary Figure 1: Representative cytospin images of proerythroblasts (Pro-E), basophilic erythroblasts (Baso-E), polychromatic erythroblasts (Poly-E), orthochromatic erythroblasts (Ortho-E), and mature erythrocytes. [file 9496058.f1.doc]
