# Supplementary material for: SOX6 Downregulation Induces γ-Globin in Human β-Thalassemia Major Erythroid Cells
Source: Biomed Res Int. 2017 Nov 28;2017:9496058. doi: 10.1155/2017/9496058 (PMC5733236; doi:10.1155/2017/9496058)
Supplement: Supplementary 2 — Supplementary Figure 2: Proportion of erythrocytes in the differentiated cultured cells on day 5, day 10, and day 15, verified by cell count based on Wright-Giemsa-stained morphology (counts of cells include Pro-E, Baso-E, Poly-E, Ortho-E, and mature erythrocytes). [file 9496058.f2.doc]

**Supplementary Figure 2.** Proportion of erythrocytes in the differentiated cultured cells on day 5, day 10 and day 15, verified by cell count based on Wright-Giemsa-


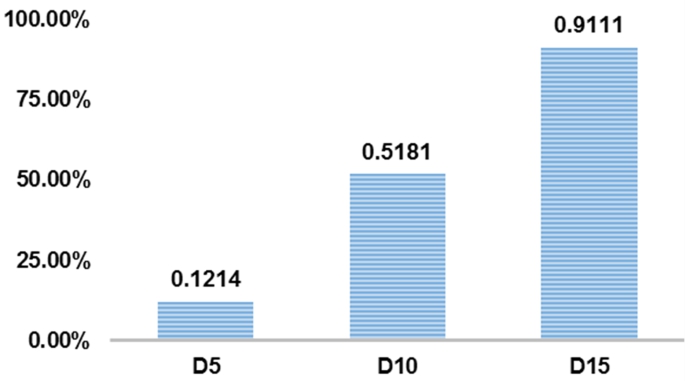
stained morphology(Counts of cells include Pro-E, Baso-E,Poly-E,Ortho-E and mature erythrocytes).
