# Supplementary material for: SOX6 Downregulation Induces γ-Globin in Human β-Thalassemia Major Erythroid Cells
Source: Biomed Res Int. 2017 Nov 28;2017:9496058. doi: 10.1155/2017/9496058 (PMC5733236; doi:10.1155/2017/9496058)

**Supplementary Figure 3.** Flow cytometry analysis showing CD235a expression in

cultured cells after 15 days.

Isotype


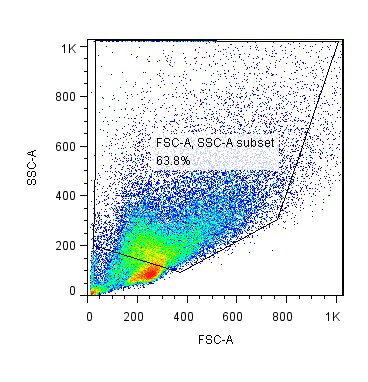

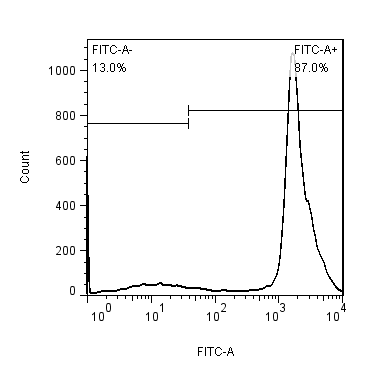


D15


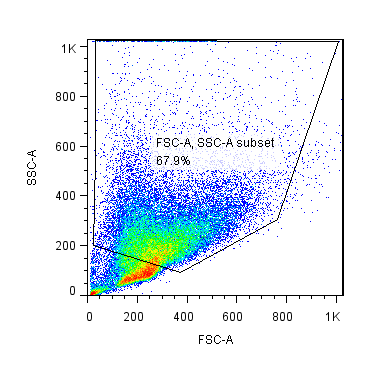

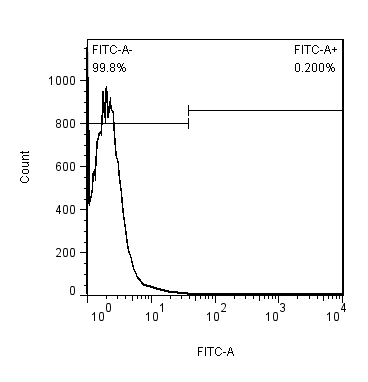

Supplement: Supplementary 3 — Supplementary Figure 3: Flow cytometry analysis showing CD235a expression in cultured cells after 15 days. [file 9496058.f3.doc]
