# Supplementary material for: SOX6 Downregulation Induces γ-Globin in Human β-Thalassemia Major Erythroid Cells
Source: Biomed Res Int. 2017 Nov 28;2017:9496058. doi: 10.1155/2017/9496058 (PMC5733236; doi:10.1155/2017/9496058)
Supplement: Supplementary 4 — Supplementary Figure 4: qPCR showing CD235a expression in differentiated cultured cells on day 5, day 10, and day 15. [file 9496058.f4.doc]

**Supplementary Figure** **4.** qPCR showing CD235a expression in differentiated cultured cells on day 5, day 10 and day 15.


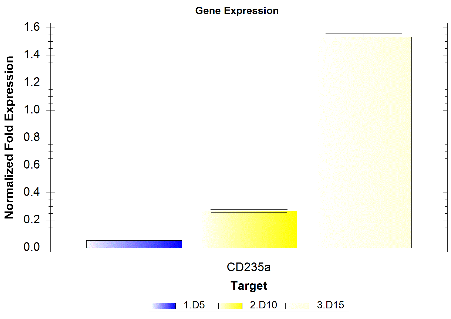


| CD235a  Expression | D5 | D10 | D15 |
| --- | --- | --- | --- |
|  | 0.052±0.00091 | 0.268±0.011 | 1.533±0.023 |
