# Supplementary material for: SOX6 Downregulation Induces γ-Globin in Human β-Thalassemia Major Erythroid Cells
Source: Biomed Res Int. 2017 Nov 28;2017:9496058. doi: 10.1155/2017/9496058 (PMC5733236; doi:10.1155/2017/9496058)
Supplement: Supplementary 5 — Supplementary Figure 5: qPCR showing γ/β-globin mRNA ratios in differentiated cultured cells on day 5, day 10, and day 15. [file 9496058.f5.doc]

**Supplementary Figure 5.** qPCR showing γ/β-globin mRNA ratios in differentiated

cultured cells on day 5, day 10 and day 15.


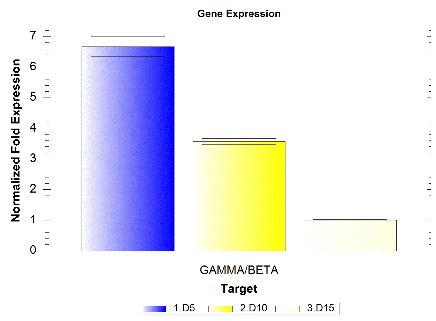


| GAMMA/BETA | D5 | D10 | D15 |
| --- | --- | --- | --- |
|  | 6.67±0.32 | 3.57±0.10 | 1±0.02 |
